# Helping medical students to acquire a deeper understanding of truth-telling

**DOI:** 10.3402/meo.v20.28133

**Published:** 2015-11-11

**Authors:** Samia A. Hurst, Anne Baroffio, Marinette Ummel, Carine Layat Burn

**Affiliations:** 1 Institute for Ethics, History, and the Humanities, Geneva University Medical School, Geneva, Switzerland; 2 Unit of Development and Research in Medical Education, Geneva University Medical School, Geneva, Switzerland; 3 CURML – University Centre of Legal Medicine, Lausanne, Switzerland; 4 Nursing Department, HE-ARC, University of Applied Sciences, Neuchâtel, Switzerland

**Keywords:** clinical education, ethics and humanities, communication skills

## Abstract

**Problem:**

Truth-telling is an important component of respect for patients’ self-determination, but in the context of breaking bad news, it is also a distressing and difficult task.

**Intervention:**

We investigated the long-term influence of a simulated patient-based teaching intervention, integrating learning objectives in communication skills and ethics into students’ attitudes and concerns regarding truth-telling. We followed two cohorts of medical students from the preclinical third year to their clinical rotations (fifth year). Open-ended responses were analysed to explore medical students’ reported difficulties in breaking bad news.

**Context:**

This intervention was implemented during the last preclinical year of a problem-based medical curriculum, in collaboration between the doctor–patient communication and ethics programs.

**Outcome:**

Over time, concerns such as empathy and truthfulness shifted from a personal to a relational focus. Whereas ‘truthfulness’ was a concern for the content of the message, ‘truth-telling’ included concerns on how information was communicated and how realistically it was received. Truth-telling required empathy, adaptation to the patient, and appropriate management of emotions, both for the patient's welfare and for a realistic understanding of the situation.

**Lessons learned:**

Our study confirms that an intervention confronting students with a realistic situation succeeds in making them more aware of the real issues of truth-telling. Medical students deepened their reflection over time, acquiring a deeper understanding of the relational dimension of values such as truth-telling, and honing their view of empathy.

Truth-telling is central to doctor–patient communication. Along with the expanding patient-centred care approach (1), attitudes and practices of truth-telling have substantially changed over time, shifting from whether, to when, and how to tell the truth to patients in clinical practice (2, 3). The decision-making process includes the consideration of the shared power and control between doctors and patients, as well as the degree to which the doctor should share information with the patient (4). Truth-telling is considered as an important component of respect for patients’ self-determination and is usually preferred by patients themselves even in the case of bad news (5).

In the context of breaking bad news, however, truth-telling is also a distressing, difficult (6), and in rare cases even a dangerous task, which can go devastatingly wrong if conducted with insufficient skill or care (7). Breaking bad news is not only about truth-telling: it must also incorporate elements such as how to manage patient emotions, how to use the adequate communication skills, and how to anticipate consequences of the disclosed information on the patient and his or her family members (8, 9). A recent metasynthesis identified that physicians in a situation of breaking bad news need to adapt to various factors related to the micro-system and the macro-system: their relationship to the patient, the patient's family, the institutional context, and the cultural milieu (10).

Truth-telling does, however, remain a central component of breaking bad news. It is a complex task requiring multiple skills in communicating, understanding, and empathising (11). Consequently, its teaching has been included in pre- and postgraduate medical curricula, first in medical ethics programmes, then increasingly in communication skills training (12). At the Geneva University Medical School, we have attempted integration of ethics with communication curricula in teaching truth-telling. Students follow a longitudinal programme of ethics teaching, running from the first (pre-clinical) to the fifth (clinical) year of the curriculum, progressing from basic concepts, to increasingly concrete application, to realistic cases. Initially, the ethical concept of truth-telling was taught through a case-based seminar, exploring the values involved in disclosure or non-disclosure of information to a patient. This format, however, did not enable students to sense the difficulty of the exercise. Based on the evidence from a literature review, the team decided to introduce an experiential learning approach aimed at providing students with the opportunity to practise ethical concepts in a realistic and safe situation, to receive an immediate and multisource feedback, and to critically reflect on the experience (13, 14). The central goals of this intervention were to introduce students to the ethical reasons for truth-telling as well as to the required reasoning and communication skills, through an exercise involving breaking bad news. This intervention used small group teaching with a simulated patients (SPs) methodology (15–17). Teaching with SPs supports and enhances a patient-centred approach during a medical interview (1, 15, 18) and gives a unique opportunity to integrate the patient's perspective in developing communication skills and other facets of professionalism (16, 19). In addition, the SP can be trained to provide feedback to trainees and bring the patient's expectations, preoccupations, feelings, and disease consequences in daily life (19) to the trainees’ attention. After the SP feedback, a debriefing is done with faculties aimed at integrating communication and ethical skills. Feedback from SPs, faculty, and peers reflected as a group on what information was critical, and on the ethical aspects of truth-telling (13, 14).

Teaching interventions using experiential methods, such as SP methodology and the principle of learner-centred learning approach (20–22), can be effective and can result in a significant increase in learners’ self-reported comfort, confidence, and self-efficacy in breaking bad news (20, 21, 23–25). In addition, recent data suggest that it also improved students’ skills in breaking bad news based on raters’ observation after SP interaction (26). Although this mode of teaching appears efficient, only its short-term effect on competency has been studied. Longitudinal surveys, assessing potential changes of skills in a situation of breaking bad news, are scarce. In particular, little is known regarding how such teaching interventions prepare students to deal with real-life situations; nor do we know how students integrate what they have learned with experience acquired during clinical rotations. Moreover, there is, to our knowledge, no report on the effect of a SP-based intervention on medical students’ ethical attitudes. To explore these questions, we designed a longitudinal study, with both quantitative and qualitative components, aimed at investigating the long-term effect of our SP-based intervention on students’ ethical attitudes towards truth-telling and perceived competence and comfort with truth-telling in the context of breaking bad news (27). Our quantitative findings suggested that students’ ethical attitudes towards truth-telling remained stable, they developed new skills following the intervention, and they also increased their awareness of the difficulties and challenges raised by the situation of breaking bad news. In this paper, we report complementary qualitative findings on medical students’ reported ethical and communication difficulties with truth-telling in the context of breaking bad news.

## Participants and methods

### Participants

At the time of the intervention, preclinical third year students (out of 6 study years) were recruited (120 in 2004 and 105 in 2005), and the two cohorts of medical students were followed through their clinical rotations (fifth year).

### Teaching intervention

The teaching intervention was a 90-min, SP-based seminar jointly developed by the ethics and clinical communication teams. It included a 15-min ethical discussion on truth-telling and a 60-min practice of communication skills in the context of a breaking bad news case. The learning objectives were (1) to provide students with an opportunity to experience the application of ethical concepts to a realistic situation and (2) to integrate doctor–patient communication skills and ethical skills in balancing what to tell and how to tell.

The SP scenario portrayed a young female pianist with an initial episode of multiple sclerosis, who was now asymptomatic although laboratory and imaging analyses confirmed the diagnosis. The choice of this clinical situation aimed at giving students a scenario which included uncertainty about the future, thus increasing the ethical challenges concerning truth-telling. The SPs’ emotional responses to the bad news included shock and denial. The SP scenario was developed to require minimal in-depth medical knowledge. Three SPs were trained for this role.

The intervention involved a group of up to 10 students, and was facilitated by a tandem consisting of a specialist in ethics and a clinician. They were first informed of the session's objectives, and they received key medical information needed to answer the SP's medical questions. Then, each student in turn conducted a sequence of the medical interview, observed by the rest of the group. Between each sequence, short debriefings allowed facilitators to provide feedback, to guide students, and to reflect on alternative techniques to practise. The SPs gave their feedback at the end of the session. Thus, the debriefing could benefit from a multi-source perspective, supporting the evolvement towards better student performance and patient satisfaction.

### Data collection

We constructed a survey instrument, including both closed-ended and open-ended items. Questionnaire items were based on published surveys (23, 28, 29). Published items were translated into French and back translated into English for quality control. Socio-demographic questions such as gender and previous participation in the teaching intervention were integrated. Survey items are further described elsewhere (27). In all questionnaires, open-ended questions addressed medical students’ concerns with the breaking bad news exercise. Students were asked ‘What concerns do you have regarding breaking bad news to patients? Please give an open answer’. Students were also asked for comments.

The study was conducted from October 2004 to May 2008. Participants answered the survey three times during medical ethics seminars: 1 week before the intervention (survey 1), 1 month after the intervention (survey 2), and 2 years later during clinical rotations (survey 3).

### Protection of human participants

Students’ participation in the research was voluntary and anonymous. The Chair of the Public Health Research Ethics Committee in Geneva designated this study exempt from ethical review. To respect autonomy, students received the information that they were free to participate in the study. We considered the students’ responses to the questionnaires as the consent to participate in the study. Confidentiality was assured by using a self-generated unique non-identifying code for each student to match responses from each student across the duration of the study.

### Data analysis

Responses to open-ended questions were transcribed and imported into QSR NUD*IST, version N6 (QSR International, Victoria, Australia), qualitative software to facilitate data analysis and allow quantification of results. Codes for participants’ concerns regarding delivering bad news, and their comments, were developed through coding of all content by the authors (SAH, CLB, AB, and MU) as a group, with regular discussions to resolve disagreements, refine, and group the content into first-level categories. All authors then used the resulting coding grid to recode the entire content in parallel, with regular meetings to resolve disagreements. Finally, we conducted a matrix analysis to compare the most salient concerns related by students at different stages of their studies. Quotations presented in this article are illustrative of the identified phenomena and are translated from the original version in French.

## Results

### Respondents

The 2004 (*n*=120) and 2005 (*n*=105) cohorts of third-year medical students were invited to participate in three sequential surveys for this study. From the 225 students, 164 (73%) took the survey before the teaching intervention, 150 (67%) after the intervention, and 114 (51%) during their fifth year of study during clinical rotations. Females represented 55% of the total. During clinical rotations, nine students declared they themselves had delivered bad news, 85 had observed their resident doing it, and 20 students had never been in the situation.

### Students’ comments

The seminar was viewed as useful, even when one of its effects was to make the student more aware of the difficulty of breaking bad news:

An excellent seminar which succeeds in making the student very uncomfortable and this is very good. More such seminars ought to be organized. (Respondent 94, survey 2)

### Medical students’ concerns with breaking bad news

Students expressed concerns about the communicational, ethical, emotional, and practical aspects of breaking bad news. These are outlined in Table 1. The most frequent concerns focused on difficulties with communication, which were expressed by 87, 68, and 63% of students before the intervention, after the intervention, and following clinical experience, respectively. Concerns regarding emotional aspects of breaking bad news were expressed by approximately half of the respondents at all three stages (58, 52, and 48%). Specifically expressed emotions included anxiety and general emotional discomfort; fear of sadness, of uncontrollable laughter, of the unknown or having an unexpected emotional reaction; and embarrassment. Ethical concerns decreased after the intervention and were again expressed more frequently after clinical experience (50, 28, and 36%). Practical concerns were also expressed, though less frequently (13, 11, and 15%).

**Table 1:** Medical students’ concerns regarding breaking bad news

	Respondents (N=225)		
	
	Before intervention (144)	After intervention (115)	Clinical rotations (95)
Communication concerns	125	78	60
Choosing words	67	38	27
Appropriate behaviour	58	35	17
Adapting to the patient	48	18	14
Supporting the patient	21	17	15
Empathy	14	7	11
Managing time	11	12	7
Lacking competence	13	9	7
Appropriate distance	17	8	2
Adapting to the situation	10	1	3
Maintaining the doctor–patient relationship	8	5	5
Managing the discussion	3	6	1
Knowing oneself well	1	1	0
Being victim of a misunderstanding	0	0	1
Family pressure	0	0	1
Emotional concerns	83	59	45
Facing the patient's emotions	55	41	30
Specific expressed emotions	24	12	5
Managing one's own emotions	17	19	17
Emotional distance and personal implication	17	9	11
Feeling powerless	7	6	7
Empathy is difficult	3	1	3
Emotions of patient's family	3	1	1
Being associated with the bad news	3	3	0
Feeling of injustice	1	0	0
Ethical concerns	71	32	34
Improving consequences for the patient	50	22	20
Truthfulness	20	6	7
Respecting the patient	11	4	8
Personal responsibility	11	3	4
Respecting autonomy	5	1	0
Doing one's best	4	4	1
Integrity	2	0	3
Not acting like a member of the family	1	0	0
Not remaining technical	0	0	2
Being fair	0	0	1
Practical concerns	18	12	14
Allowing treatment to take place	9	5	6
Not having enough experience	3	2	4
Obtaining long-term follow-up	2	2	0
Having time for the patient	1	3	4
Including the patient's family	3	0	0
Finding oneself in a difficult position	1	1	0
Announcing uncertainty is difficult	0	1	0

### Evolution of medical students’ concerns through time

As the frequency of medical students’ concerns changed over time, so did the nature of these concerns as reflected in their responses. We examined the most frequently expressed concerns and examined how they were presented at the three stages of respondents’ training. As the students progressed, new expressions of the same concerns emerged. Representative citations are presented in Table 2.

**Table 2:** Integrating values and goals through time

	Before intervention	After intervention	Clinical rotations
Communication concerns			
Choosing words	Avoid direct wording	Being clear	Being balanced
	*The fear of being too brutal by not using the appropriate words*. (Respondent 8)	*It's difficult to be able to transmit all the necessary information in difficult circumstances*. (Respondent 204)	*To not reassure the patient too much with false hopes*. (Respondent 109)
Appropriate behaviour	Inexperience	Clumsiness	Support
	*I feel that I do not have the necessary tools to give this information in the best possible way*. (Respondent 18)	*Being clumsy in my remarks and so harm the patient*. (Respondent 20)	*My first preoccupation is to be appropriate so that the patient feels supported*. (Respondent 284)
Adapting to the patient	Predictive planning	Support in situ	Lifelong adaptation
	*Knowing how they will react, how they will come to terms with the news at the time and most of all afterwards, when we – the doctor – are not there any more*. (Respondent 231)	*It's impossible to predict the reaction the patient will have (…). However, it is reassuring to know that we will do all we can to assist them in their distress*. (Respondent 15)	*I think it is easier (…) when we practice medicine for some years (…). It is never possible to predict a patient's reactions (so) we must be capable of adapting to him, his life experience, and his wishes*. (Respondent 201)
Supporting the patient	The goal of comforting	The goal of comforting	Practical examples
	*Being able to comfort the patient as much as possible, while giving her the time to come to terms with the information*. (Respondent 215)	*To alleviate her distress*. (Respondent 209)	*It is difficult to wait, we want to continue. But it's best to wait for the reaction and, if needed, to take out a handkerchief or offer a comforting shoulder*. (Respondent 72)
Empathy	An emotion	A perception tool	A clinical skill
	*So, I think it's really a selfish reason, because I may have too much empathy and it hurts me to have to announce bad news*. (Respondent 257)	*I do not know what attitude to have; I have a hard time feeling the patient's feelings*. (Respondent 40)	*Being able to ‘put yourself in the patient's skin’, to understand him*. (Respondent 69)
Lacking competence	Lack of general knowledge	Lack of specific knowledge	Lack of embedded knowledge
	*My main concern is not being able to deal with the reactions and questions of the person to whom we give bad news*. (Respondent 27)	*A very good (i.e., perfect) knowledge of the disease and situation is necessary so as not to be embarrassed by the patient's questions*. (Respondent 208)	*Not knowing the diagnosed pathology well enough and making errors in prognosis, quality of life, which could have bad consequences for the patient*. (Respondent 65)
Managing time	Preparing the patient	Managing the encounter	Giving the patient time
	*It is difficult to succeed as well as possible in preparing the patient to hear the bad news*. (Respondent 214)	*The fear of being too moved myself to manage the timing of the discussion*. (Respondent 24)	*Sometimes it is difficult to wait, because we want to carry on. But the best thing is to wait for the patient's reaction*. (Respondent 72)
Maintaining the doctor–	The patient could leave	The doctor could harm the link	Dialogue could end
patient relationship	*A risk of the patient severing the link, or loosing trust*. (Respondent 82)	*We have to manage the moment, not downplay the bad news, but try to do it well and not lose the contact with the patient*. (Respondent 10)	*Keep a relation of trust and not end the dialogue*. (Respondent 104)
Emotional concerns			
Facing the patient's	Understanding the patient's emotion	Helping the patient through the emotion	Staying in synch with the patient
emotions	*I hope to understand their emotions a little in order to be able to help better with the bad news*. (Respondent 7)	*(Fear of) the patient's revolt, and not being able to calm or comfort him*. (Respondent 77)	*To not be in the right ‘state of mind’, for example to be happy for a personal event and not be able to remain grave*. (Respondent 65)
Managing one's own	Setting one's emotions aside	Tuning one's own emotions down	Avoiding emotional contagion
emotions	*To be unable to hide my own emotions in front of the patient*. (Respondent 3)	*It is very difficult to manage a situation like this one when you are submerged by your own emotions*. (Respondent 208)	*I fear entering into sympathy with the patient and becoming destabilized by my own emotions*. (Respondent 206)
Emotional distance and	A step back	Being available	The ‘right’ distance
personal implication	*(Fear) of taking the bad news personally and too much to heart, and not stepping back from these situations enough*. (Respondent 15)	*Knowing how to be present without overwhelming the patient*. (Respondent 99)	*To place enough distance from the patient while remaining human*. (Respondent 205)
Ethical concerns			
Improving consequences	Not making the situation worse	Avoiding bad consequences	Sustaining the patient
for the patient	*To do it in the right manner, so that it does not make the bad news even more difficult than it already is*. (Respondent 29)	*The patient's reactions can be very violent, I'm thinking in particular of suicidal thoughts*. (Respondent 287)	*Encourage him despite it all (to fight, to keep going) without giving him false hopes*. (Respondent 211)
Truthfulness	Hiding nothing	A truthful evaluation	A truthful evaluation
	*Being capable of explaining as honestly and completely as possible. (Respondent 47)*	*Being understood from the start, without giving false hopes or presenting a situation more dramatic than the truth*. (Respondent 9)	*To not reassure the patient falsely, nor falsely increase his despair and fear*. (Respondent 60)
Respecting the patient	Not imposing one's views	Adjusting to the patient	Adjusting to the whole patient
	*I hope to respect the beliefs or culture of patients, without imposing mine in announcing bad news*. (Respondent 7)	*Responding with the right touch and tact to the questions asked by the patient*. (Respondent 78)	*(Fear of) not responding to his expectations as a whole person (culture, character, etc…)*. (Respondent 105)
	*Some patients give us to understand that they do not want to know, others do the opposite*. (Respondent 16)		

#### Students, patients, relationships

Over time, several of the concerns expressed shifted from a personal focus on the student to a relational focus on what the patient was experiencing and how the student ought to adapt to it. Concerns focused on ‘choosing words’, gradually shifted from the emission of information to their reception by the patient and her ability to integrate them. Those focused on ‘managing time’, ‘appropriate behaviour’, ‘maintaining the physician–patient relationship’, ‘facing the patient's emotions’, ‘emotional distance and personal implication’, ‘improving consequences for the patient’, as well as ‘respecting the patient’, also shifted from a focus on what the student had to do, to what the patient was doing or experiencing, and then sometimes to an interaction between the student and the patient or more generally to the relationship itself.

#### Awareness of what is being learned

Concerns related to ‘adapting to the patient’ followed a different course. Prior to the intervention, students expressed a concern about learning how to predict a patient's reaction in order to better help the patient through a difficult situation. Following the intervention, they realised that this was not possible, and concerns for adaptation focused on supporting the patient once the bad news had been announced and the patient's reaction became visible. After clinical experience, students who voiced such concerns tended to realise that this would continue to require an effort of adaptation to new patients throughout their lives and would never follow a completely acquired set of scenarios.

Concerns related to ‘supporting the patient’ and ‘managing the students’ own emotions’ became more concrete over time, for example, with practical examples of how students would offer comfort appearing after clinical experience.

#### Truthfulness

Concerns for ‘truthfulness’ were expressed mostly in the first and third questionnaire, as ethical concerns in general were voiced less frequently immediately after the intervention. Before the teaching intervention, ‘truthfulness’ mostly meant a concern to hide nothing from the patient. After the intervention and after clinical experience, medical students’ concerns with ‘truthfulness’ shifted to mean avoiding false hopes and maintaining a truthful balance between positive and negative aspects of the situation. ‘Truthfulness’ thus shifted its meaning to become about helping the patient to attain a true evaluation of his or her situation.

#### Empathy

The different meanings of ‘empathy’ and their progression through time also reflected a progression of their view of the patient as medical students gained experience. They progressed from a view of empathy as emotional contagion, to a view of empathy as a perception tool to help them understand their patients’ emotions and their understanding of the situation.

### Components of truth-telling

Truthfulness, choosing the right words, empathy, managing the patient's and student's emotions, and adapting to the patient were all associated by students with components of truth-telling. Where truthfulness was a concern for the content of the message, truth-telling included broader concerns regarding how information was communicated and how realistically it was received. This included concerns about avoiding brutal wording or any formulation that could overwhelm the patient and hinder understanding. In this sense, truth-telling required empathy, adaptation to the patient, and appropriate management of emotions not only out of a concern for the patient's welfare but also in order to ensure realistic understanding of the situation.

(My concerns are) still the same: to find the right words to tell the truth without ill-treating the person and also managing to temper her pain. (Respondent 209, survey 2)

## Discussion

Our study provides insight into medical students’ progress with the integration of different components of truth-telling throughout their studies (27). While the emotional impact of the teaching intervention tended to displace ethical concerns, these were expressed again after clinical experience. At that time, their content had shifted from a personal focus on the student to a relational focus on the interaction between the student and the patient.

In support of our quantitative findings, suggesting that the teaching intervention increased students’ awareness of the difficulties and challenges raised by a situation of breaking bad news (27), these qualitative results confirm that this intervention succeeded in making some students more uncomfortable with breaking bad news (27). That students reported feeling less prepared after the intervention than before it, might represent both an unmet need for help in managing this discomfort and a missed opportunity for providing students with more practical and communication tools at a time when they could be particularly receptive. It was, however, identified as a positive outcome and perceived as useful for this reason. That students expressed concerns which integrated communicational and ethical components suggests that our attempt at integrating the practice of communication skills and ethical reflection in a realistic situation was successful, but a longer follow-up may be required.

Students were reflective to begin with, as illustrated by elaborate comments on the initial questionnaire, but they deepened their reflection following the intervention. Confrontation with an SP's emotional reaction while receiving bad news can be destabilising, but is likely to provoke reflection (13, 30). Supportive input from facilitators aimed to enable students to reflect on their actions by connecting theory and practice (31) and by challenging underlying assumptions and considering new perspectives. Feedback from peers, teachers, and SPs aimed to allow students to reflect on that feedback and gain insights into areas such as empathy and addressing patients’ concerns (32–34). Thus, the benefits of reflection may rely on an appropriate setting of the intervention as well as on supervisor support (31). In analysing longitudinal follow-up, distinguishing the effects of this seminar from that of students’ growing clinical experience remains difficult. One goal of our intervention, however, was to make students more aware of the learning objectives associated with ethical and communicational aspects of truth-telling. Our quantitative data suggests that this was successful (27), and this may also have helped students to be more receptive to learning from their clinical experiences.

The evolution of students’ comments in our study revealed a decrease in the number of concerns expressed after the intervention and a shift from a personal focus on the student to a relational focus on the interaction between the student and the patient. In particular, the salience of concerns related to ‘using the appropriate words’, ‘student's ability to manage the patient's emotions’, ‘adapting to the patient’, and ‘balancing emotional involvement’ decreased post-intervention. This finding partly confirms those obtained in a different setting by Rosenbaum and Kreiter (23). In contrast, this was not true for the salience of concerns related to ‘managing the students’ own emotions’. Several reasons can be hypothesised. Students may have fewer concerns after an intervention designed to help them address some of them. They have been reassured by an intervention calibrated to present them with a manageable level of difficulty. Alternatively, they may be focusing more directly on what they now view as the main concerns after having experienced a difficult situation that they could only imagine before. Finally, they may be discouraged by the intervention and voice fewer concerns because they have become more fatalistic. We did not, however, note any comments from participants in support of the last hypothesis. The shift from a personal to a relational focus is striking in the different concerns expressed by students. This suggests that students could indeed experience the dynamic and patient-dependent aspects of truth-telling (2). This constitutes an important dimension of learning the patient-centred care approach (1) and of decision-sharing in particular (4).

As we reported previously (27), ethical attitudes towards truth-telling remained stable throughout the study. This apparently contradicts other studies identifying erosion of moral reasoning and empathy in general, an effect that has been interpreted as an interruption of medical students’ moral growth (22, 35–38). Empathy, in particular, has been reported as endangered by medical studies, especially by the experience of clinical years (39, 40). Clinical workloads, emotional suppression as a self-protection strategy, as well as role modelling by non-uniformly empathic clinical teachers have been proposed as possible factors. Data suggesting geographic variation in the evolution of empathy, with Japanese, Ethiopian, and Portuguese medical students’ empathy increasing during their studies (41–43), may support some of these hypotheses, as working conditions, approaches to emotional self-protection, and role models are likely to vary with location and culture (44). Other studies suggest that teaching interventions can be effective in preventing this erosion and in maintaining ethical sensitivity, growth, and empathy (45). Our qualitative findings suggest another hypothesis and that something additional may be at play. Ethical attitudes of medical students could indeed change, but this could be a qualitative change not measured by quantitative tools or even by methods based on stage identification such as the Defining Issues Test (46). Our results suggest that medical students do progress in their medical training, but that this takes the form of acquiring a deeper understanding of the relational dimension of values such as truth-telling and a honing of their view of empathy (39–43). They initially viewed empathy as a form of emotional contagion or compassionate communication, and later viewed it as a perception tool to help them understand their patients’ emotions and their understanding of the situation. Interestingly, empathy is also diversely defined in the literature as taking the patient's perspective, compassionate care, patient-centred care, or as something akin to emotional intelligence (47). This should caution us to treat results reporting a decrease of empathy during medical school with some care. A substantial part of the influential tools such as the Jefferson Scale of Physician Empathy consists of items reporting attitudes towards the importance of empathy, defined as a construct with different components (48). If fewer components of the construct were recognised as parts of empathy as students matured, the resulting decrease would sometimes reflect neither a decrease in the importance of empathy as understood by these students nor in their ability to integrate empathy in their work. Rather, it could reflect their increased focus on the parts of the construct which they recognised as more relevant. Studies examining more precise scores of empathy as ‘heart-reading’ or ‘mind-reading’ reported an increase in cognitive empathy during medical training in Ethiopia (43) and stable values for cognitive empathy with a small decrease in emotional empathy in male students in the United Kingdom (49). Such findings support the interpretation proposed here.

Finally, truth-telling was viewed as requiring empathy, adaptation to the patient, and appropriate management of emotions not only out of a concern for the patient's welfare but also in order to ensure realistic understanding of the situation. Caring for the patient's welfare is often contrasted to the requirement of truthfulness in the context of breaking bad news.^1^ One reason is that information is sometimes viewed as harmful to patients and the values of truthfulness and beneficence as in tension with each other (50). Moreover, this contrast serves to remind professionals that a patient who has been given bad news will need to be cared for in order to help him or her deal with a difficult situation (9, 10). In contrast, our findings suggest that caring for the patient's emotions is also a component of truth-telling: unless he or she is in a position to understand and integrate the disclosed information, simple disclosure of true information will not be sufficient for truthfulness.

Our study has several limitations. Students answered questionnaires during ethical teaching sessions, which could constitute a bias towards more attention to ethical aspects. As our response rate was high, however, such effects are unlikely to be large. As in other questionnaire studies, a bias could exist towards obtaining socially accepted answers. We tried to reduce this bias by guaranteeing complete confidentiality regarding respondents’ identity and their answers through the use of a unique respondent-generated code. As outlined above, in analysing longitudinal follow-up it remains difficult to parse out the effects of this single seminar from that of students’ growing clinical experience. Finally, as with any exploratory single-centred study, any generalisation to other contexts should be cautious.

## Conclusions

Our study confirms that an intervention confronting students with a realistic situation succeeds in making them more aware of the real issues of truth-telling. Confirming our quantitative findings, students report feeling less comfortable in breaking bad news and thoughtfully comment about why the intervention was helpful to them. Conjointly, they realise that truth-telling integrates not only ethical aspects but also communicational and emotional components. While acquiring clinical experience, students’ concerns shifted from a personal to a relational focus. Empathy, which was initially viewed as a kind of compassionate communication, evolved towards a tool for patients’ mind- and heart-reading, allowing students to tailor the information to be given according to patients’ preferences. Experiential learning and clinical experience thus contribute to students’ progress with the components of truth-telling.

## Footnotes

1 We thank the anonymous reviewer for encouraging us to develop this point.
